# 贝伐珠单抗治疗非小细胞肺癌脑转移的研究进展

**DOI:** 10.3779/j.issn.1009-3419.2016.08.05

**Published:** 2016-08-20

**Affiliations:** 030013 太原，山西医科大学附属山西省肿瘤医院呼吸科 Department of Pulmonary Oncology, Shanxi Cancer Hospital, Shanxi Medical University, Taiyuan 030013, China

**Keywords:** 肺肿瘤, 脑转移, 贝伐珠单抗, Lung neoplasms, Brain metastases, Bevacizumab

## Abstract

脑部是肺癌最常见的远处器官转移部位之一, 肺癌脑转移的发生率高, 是脑转移性肿瘤中最常见的类型, 同时也是肺癌死亡率居高不下的原因之一。抗肿瘤血管生成治疗已成为肺癌治疗的重要手段之一, 抗血管生成药物贝伐珠单抗也成为继全脑放疗、立体定位放疗和化疗之后肺癌脑转移患者新的治疗选择。目前, 对脑转移的非小细胞肺癌患者应用贝伐珠单抗治疗的临床研究也越来越多, 其安全性和有效性是研究重点。本文就贝伐珠单抗治疗非小细胞肺癌脑转移的研究进展做一综述。

## Abstract (en)

Brain metastases are frequently encountered in patients with non-small cell lung cancer (NSCLC) and are a significant cause of morbidity and mortality.Antiangiogenesis therapy plays a major role in the management of brain metastases in lung cancer.Bevacizumab have become the novel method for the treatment of lung cancer with brain metastases beyond the whole brain radiation therapy, stereotactic radiosurgery and chemotherapy.Recently, more and more studies and trials laid emphasis on the bevacizumab for NSCLC with brain metastases treatment.The key point is the efficacy and safety.In this review, bevacizumab therapy of NSCLC with brain metastases were summarized.

肺癌是全球最为常见的恶性肿瘤之一, 非小细胞肺癌(non-small cell lung cancer, NSCLC)约占所有肺癌的80%, 其中30%-43%的患者在疾病进展过程中出现脑转移, 肺腺癌患者脑转移超过50%^[1]^。脑转移治疗常用方法是放疗、手术治疗及支持治疗, 但即便经过积极治疗, 预后依旧较差^[2]^, 因此需要更为有效及系统性的方案来控制NSCLC患者的脑转移^[3, 4]^。近年来研究发现抗血管内皮生长因子(vascular endothelial growth factor, VEGF)单克隆抗体贝伐珠单抗在抗血管生成方面对NSCLC脑转移治疗有效。既往研究出于对脑出血的担忧, 贝伐珠单抗的前期临床试验排除了脑转移患者, 但随后多项临床试验进一步研究结果显示, 贝伐珠单抗在有脑转移的NSCLC患者中显示出较好的安全性和有效性, 并不会增加中枢神经系统出血的发生率^[5, 6]^。鉴此, 2009年欧盟取消了贝伐珠单抗的说明书限制, 将未经治疗的脑转移患者纳入贝伐珠单抗的适应症。因此肺癌美国国立综合癌症网络(National Comprehensive Cancer Network, NCCN)指南在讨论部分提出:贝伐珠单抗可用于中枢神经系统转移灶已在治疗的患者。目前对脑转移的NSCLC患者应用贝伐珠单抗治疗的研究逐渐增多。本文就贝伐珠单抗治疗NSCLC脑转移的研究进展做综述。

## 1 NSCLC脑转移的血管生成机制

NSCLC脑转移的发生是一个高度选择性、多步骤的过程, 有赖于肿瘤与宿主细胞的相互作用。转移性癌进入脑循环后, 停留在血流缓慢的毛细血管床, 它们和内皮细胞相互作用并以粘附分子作为媒介穿过内皮细胞。一旦攻克血脑屏障, 癌细胞将面对细胞外基质、局部的脑实质细胞如星行细胞和小胶质细胞, 副分泌信号分子如细胞因子等。肿瘤的生存和生长需要适应这些因子并与之相互作用。同时脑组织通过血管内皮因子、血管扩张和血管生成模拟机制“帮助”转移瘤生产繁殖^[7]^。在上述过程中, 血管生成与脑转移的发生发展密切相关。

肺癌脑转移灶中的血管生成可以通过多条通路, 而VEGF信号通路是最主要也是研究最多的信号通路。研究证实脑转移依赖VEGF, VEGF在脑转移灶中呈高表达与预后不良相关^[7]^。VEGF不仅能够诱导新生血管形成还能影响血管的通透性。新生血管不具备完整的血脑屏障, 为肿瘤细胞的迁移和扩散提供了有利的条件; 另一方面, 血管内皮不完整, 通透性增加, 导致液体和血清蛋白渗出, 形成瘤周水肿^[8]^。瘤周水肿的危害有三个方面:首先, 增加肿瘤占位体积, 导致或加重神经功能障碍, 引起或加重颅内压增高症状; 其次, 分离肿瘤和正常组织, 为肿瘤提供生长空间, 导致局部灌注不良, 阻滞药物进入、引起代谢产物堆积, 最终导致局部出现缺氧和酸中毒, 缺氧和PH值降低是诱导VEGF过表达的重要因素, 从而形成恶性循环。最后, 瘤周水肿会增加手术风险和放疗早期损伤。基础研究^[9]^发现抑制VEGF信号通路可有效降低动物模型中脑转移的发生率, 同时回顾性的临床研究^[10]^显示贝伐珠单抗联合化疗较单独化疗可有效降低晚期NSCLC脑转移的发生。

## 2 贝伐珠单抗的作用机制

贝伐珠单抗是一种重组的人类单克隆IgG抗体, 可以特异性的与VEGF结合, 阻断VEGF和其受体的结合, 从而减少新生血管生成, 诱导现有血管的退化, 抑制肿瘤生长。这种拮抗VEGF的效应可以抑制不成熟的血管生成, 诱导血管正常化, 从而增加肿瘤内部的灌注, 增加药物输送率。Fischer等^[11]^研究发现经贝伐珠单抗治疗后的高级别胶质瘤患者均出现肿瘤内部的微血管密度较治疗前降低, 血管的形态在贝伐珠单抗治疗后出现血管正常化, 肿瘤血管功能得到改善, 药物输送率增加, 上述观点得到进一步证实。另一方面, 贝伐珠单抗还可以通过有效控制NSCLC脑转移灶周围水肿, 提高患者预后。借鉴贝伐珠单抗治疗胸腔积液、高级别胶质瘤经验^[12]^, 将贝伐珠单抗用于治疗NSCLC脑转移灶周围脑水肿, 由于VEGF过表达是瘤周水肿形成的重要机制, 所以抗VEGF治疗能够有效的控制瘤周水肿。多个病例报道和临床研究均显示, 贝伐珠单抗能够有效的缓解瘤周水肿引起的症状和神经功能障碍, 降低糖皮质激素的使用量, 降低影像学显示的水肿程度。Zustovich等^[13]^报道, 以贝伐珠单抗为基础联合吉西他滨+卡铂治疗NSCLC脑转移患者的初期临床数据显示, 经治疗后患者无进展生存期(progression free survival, PFS)达到9.1个月, 总生存期(overall survival, OS)达到9.6个月, 而且经治疗的13例患者均未出现颅内出血、脑血管意外事件及严重高血压等不良反应, 经治疗患者的脑水肿也消失了。

## 3 贝伐珠单抗的安全性和疗效

### 3.1 贝伐珠单抗联合化疗

近年来多项临床研究表明, 以贝伐珠单抗为基础联合化疗治疗晚期不可切除的NSCLC脑转移不仅安全可耐受, 而且疗效明显。Socinski等^[14]^在PASSPORT研究中发现患者一线使用贝伐珠单抗(每3周使用1次)联合铂类为基础的双药方案或厄洛替尼, 二线治疗中采用贝伐珠单抗联合单药化疗或厄洛替尼治疗至疾病进展或死亡, 采用贝伐珠单抗治疗的106例脑转移的患者均未出现2级以上颅内出血、脑血管意外等严重不良反应, 结果表明应用贝伐珠单抗治疗NSCLC脑转移安全且耐受。2013年Zustovich等^[15]^报道, 包括NSCLC在内的脑转移患者经过贝伐珠单抗联合化疗后, PFS达14个月, OS达15个月, 治疗所致轻度不良反应可控, 未见颅内出血等严重不良反应发生。多项回顾性研究也得到与上述相似结果^[16-18]^。

Tang等^[19]^回顾性分析了776例晚期伴有脑转移的NSCLC患者经过单纯化疗、化疗联合贝伐珠单抗、表皮生长因子受体酪氨酸激酶抑制剂(epidermal growth factor receptor-tyrosine kinase inhibitor, EGFR-TKI)和支持治疗四种不同疗法的疗效。结果显示:经化疗+贝伐珠单抗治疗的患者中位PFS高于另外三组(8.5个月:5.0个月:8.0个月:1.5个月, *P* < 0.05);中位OS高于单纯化疗和支持治疗(10.5个月:7.3个月:3.0个月, *P* < 0.01)。另外, 该研究依据EGFR突变情况进行了亚组分析, 其中EGFR野生型组(360例患者)中经化疗+贝伐珠单抗的患者中位PFS(*P* < 0.01)和中位OS(*P* < 0.01)都高于化疗和支持治疗。该试验表明化疗+贝伐珠单抗治疗晚期NSCLC脑转移有效。

BRAIN研究(NCT00800202)是一项多中心的非随机非对照Ⅱ期临床研究, 旨在评价贝伐珠单抗联合一线化疗或二线厄洛替尼治疗无症状初治脑转移的非鳞癌NSCLC的疗效和安全性。该试验一组患者(*n*=67)接受一线贝伐珠单抗联合紫杉醇+卡铂治疗, 另外一非对照组患者(*n*=24)一线铂类为基础的化疗失败后接受二线贝伐珠单抗联合厄洛替尼治疗直至疾病进展或不可接受的毒性, 试验主要终点为6个月PFS率, 次要终点为缓解率、OS、安全性, 经治疗达到了预先设定的主要终点6个月PFS标准, 原发肿瘤、颅内转移与颅外转移部位患者缓解率相似, 与历史数据相比, 颅内出血率低, 本研究的总体安全性与以往研究结果一致, 研究表明含贝伐珠单抗方案治疗NSCLC脑转移患者的安全性可接受^[20, 21]^。

### 3.2 贝伐珠单抗联合放疗

贝伐珠单抗联合放疗治疗脑转移瘤的研究也取得初步进展, 有望成为NSCLC脑转移的新的治疗方案。脑转移瘤的生长离不开新生血管生成, 其血管呈非正常方式生长, 导致瘤体内部乏氧酸中毒, VEGF抑制剂贝伐珠单抗通过使肿瘤血管正常化从而减少乏氧增加放疗敏感性。近年来法国学者们进行了一项Ⅰ期研究(即REBECA试验)^[22]^, 该多中心单组试验采用3+3剂量设计, 入选者均患有从实体瘤转移且不能切除的脑转移瘤, 患者接受递增剂量的贝伐珠单抗的3周期的治疗(分别在剂量组0、1、2分别以每2周5 mg/kg、10 mg/kg和15 mg/kg的剂量治疗), 并从15天起进行全脑放疗(30 Gy/15分次/3周)治疗。剂量组3的治疗流程是:贝伐珠单抗每2周15 mg/kg, 从15天起进行全脑放疗(30 Gy/10分次/2周)治疗。结果显示, 接受治疗的19例患者, 未见剂量限制性毒性, 未见严重毒性发生, 治疗后3个月疗效评估显示, 缓解病例共10例, 其中完全缓解1例。结果证实贝伐珠单抗联合全脑放疗用于脑转移治疗可耐受, 根据良好的安全性/疗效平衡, 可进一步评价疗效^[23]^。

## 4 小结与展望

NSCLC脑转移发生机制复杂, 与血管生成密切相关, 其非正常的血管生成影响了患者生存及治疗预后。贝伐珠单抗作为抗血管生成药物可以使血管正常化, 明显改善肿瘤内部微环境, 增加血管通透性, 抑制脑水肿, 增加其他化疗药物或放疗的疗效。随着近年来贝伐珠单抗为基础治疗NSCLC脑转移的临床研究不断进展, 其安全性和疗效不断得到认可, 但仍需进一步及大量临床研究考证。
